# Palladium Complex-Loaded Magnetite Nanoparticles as Drug Delivery Systems for Targeted Liver Cancer Therapy

**DOI:** 10.3390/pharmaceutics17081033

**Published:** 2025-08-08

**Authors:** Sara A. M. El-Sayed, Ghadha Ibrahim Fouad, Hanan H. Beherei, Mohamed R. Shehata, Mostafa Mabrouk

**Affiliations:** 1 Refractories, Ceramics and Building Materials Department, National Research Centre, 33 El-Bohouth St. (Former EL Tahrir St.), Dokki, Giza P.O. Box 12622, Egypt; sarali_87@yahoo.com (S.A.M.E.-S.); hananh.beherei@gmail.com (H.H.B.); 2 Department of Therapeutic Chemistry, Pharmaceutical and Drug Industries Research Institute, National Research Centre, 33 El-Bohouth St., Dokki, Giza P.O. Box 12622, Egypt; ghadhaibrahim@gmail.com; 3 Chemistry Department, Faculty of Science, Cairo University, Giza P.O. Box 12211, Egypt; mrshehata@sci.cu.edu.eg

**Keywords:** magnetite, metal/ligand complex, molecular docking simulation, anticancer selectivity, liver cancer, diethylnitrosamine, thioacetamide, inflammation

## Abstract

**Background/Objectives**: Liver cancer is considered one of the most dangerous types of cancer due to both the patients’ and the physician’s delay in diagnosis. Metal/ligand complexes represent antitumor drugs; however, they have several limitations such as a lack of specificity that results in damage to healthy organs. Therefore, there is a need for a material that improves specificity and decreases side effects. Magnetite nanoparticles (MNPs) show outstanding findings in the targeting and treatment of cancer-diseased organs. **Methods**: Herein, a metal/ligand palladium complex with antitumor activity was prepared and loaded onto magnetite nanoparticles for the treatment of liver cancer. The proposed structures with the lowest energy geometries were identified by density functional theory (DFT) utilizing the Gaussian09 program. Molecular docking simulation was conducted on an HP Pavilion dv6 Notebook PC equipped with an AMD Phenom™ N930 Quad processor. Afterward, the prepared nano-systems were investigated using FTIR and TEM. In vitro drug release measurement was evaluated in PBS at different time intervals. Eventually, the selectivity of these nano-systems was investigated using an animal rat model. **Results**: The results showed that MNPs with a crystalline structure and superparamagnetic characteristics (Ms = 71.273 emu/g) were created with a large surface area (63.75 m^2^/g), and they were validated to be acceptable for drug delivery applications. The palladium complex [Pd(DMEN)Cl_2_] loaded onto magnetite released highly in acidic circumstances (pH 4.5), implying that it could be employed for targeted therapy of liver cancer. **Conclusions**: In vivo investigations in a rat model of liver cancer induced by diethylnitrosamine and thioacetamide (DEN/TAA) showed that the combination of the palladium complex and magnetite demonstrated a potent anticancer therapeutic activity on liver cancer in rats, improving liver function and structure while mitigating inflammation.

## 1. Introduction

Cancer causes over 8.2 million deaths each year, making it one of the leading causes of mortality worldwide [1]. Chemotherapy is considered one of the main therapeutic methods for cancer by slowing the growth of cancer cells or destroying those cells [2]. Transition metal complexes have presented a potential solution for cancer therapy. The first innovation of the transition metal complex which helps in cancer treatment was in 1965 named cisplatin [3]. Liver malignant growth is the third driving reason for disease death around the world [4]. The significant etiologies for liver malignant growth are hepatitis B infection (HBV), hepatitis C infection (HCV), liquor, and non-alcoholic steatohepatitis (NASH) [5]. Over the last 10 years, there have been changes in the weight and etiology of liver cancer [6]. The chemotherapy drugs damage the DNA of the cells by creating a chemical bond with the DNA so they can no longer reproduce [7]. The commonly utilized chemotherapy drugs for liver cancer treatment are cisplatin and 5-FU (CRUK 2006) [7]. Cisplatin is the most well-known antitumor agent that induces apoptosis by binding to DNA purine bases and forming inter- and intra-strand DNA [8]. Following the success of cisplatin as an antitumor drug, Oxaliplatin, Satraplatin, Carboplatin, and Nedapolatin were developed.

However, those metal/ligand complexes lack specificity; they do not kill only the cancer cells but also destroy healthy cells [9,10]. Moreover, they have a lot of limitations such as destroying the immune system, hair loss, inflammation of the digestive tract, and lack of solubility, which limit their use in cancer treatment [2,9,10]. Therefore, there is a need for a solution to improve their selectivity and decrease their cytotoxicity, as well as overcome the multi-drug resistance (MDR) by cancer cells. In several studies, it was reported that researchers used nanoparticles to overcome the side effects of drugs, especially anticancer [2]. Particularly, using the nanoparticles as drug carriers began after the 1980s [11]. The nanoparticles have shown success by passing the biological barriers and delivering the drug to the cancer cell directly without affecting the healthy cells and protecting them from the side effects of the antitumor drug. Magnetic nanoparticles (MNPs) have expanded impressive interests in different areas, for example, attractive information stockpiling gadgets, ferro liquids, magnetic medication transportation, magnetic imaging, and hyperthermia treatment for tissues contaminated with dangerous development such as cancer [12]. MNPs can likewise be utilized as impetuses in various applications for which they might be disengaged from a reasonable medium by applying an outside magnetic field. Accordingly, MNPs in light of paramagnetic metal oxides, particularly iron (to be specific, MNPs have been broadly investigated in biomedical fields [13].

In addition, the treatment for hepatocellular carcinoma could be monitored in real time using magnetic resonance at the same time. If the context and breadth of their application are further refined, such a strategy could serve as an efficient backup to conventional chemoembolization when conventional embolism approaches fail. Moreover, recent research has focused on the development of MNPs as delivery vehicles to magnetically collect anticancer drugs in cancer tissue [14]. The appropriate size, low systemic toxicity, magnetic responsiveness, and significant tumor cell absorption of those nanoparticles were all confirmed. The safety of the nanoparticles has been verified by cell investigations and the hemolysis assay. High tumor cell endocytosis was the result of the targeted medication delivery by external magnet attraction. The treatment’s effectiveness was further improved by the enhanced permeability and retention (EPR) effect, which facilitated passive tumor homing of the nanoparticles. Complex magnetic nanoparticles (CMNs), which contain iron oxide MNPs and antitumor drugs adsorbed onto their surface, such as the anthracycline row anticancer doxorubicin (DR), have the potential to be an important tool in the fight against cancer, according to experimental and clinical studies carried out in the last ten years. Oxidization, chemisorption on their surface, targeted surface modification, thermolysis, and mechano-chemical activation are a few of the recognized processes used in CMN formulation. Additionally, it is known that altering the nanoparticles’ surface layer can alter their magnetic characteristics relative to their core and that this interaction will cause the nanoparticles’ physical and chemical properties to vary significantly [15].

Therefore, our research illuminated a novel targeted approach for the treatment of cancer that relies on the magnetic delivery of superparamagnetic nanoparticles to the tumor site alongside an antitumor agent (metal palladium complex). Based on the above, the combination of both metal/ligand complexes with magnetite as a drug carrier would demonstrate impressive antitumor activities through targeted delivery to organs affected by cancer disease without affecting the normal tissues. To fulfill the objective of this study, the complex structure was evaluated by FTIR, NMR, and GC mass, and the whole structure (metal/ligand complex loaded onto magnetite) was investigated by FTIR and TEM analysis to assess the complicated structure. The density functional theory (DFT) and the Gaussian09 program were used to identify the recommended structures that have the lowest energy geometries. Utilizing an HP PC (HP Pavilion dv6 Notebook pc) with an AMD Phenom™ N930 Quad, molecular docking simulation was used. Finally, the produced metal/ligand complex MNPs were further tested for the treatment of liver cancer-induced rats.

## 2. Materials and Methods

### 2.1. Materials

The supplier of anhydrous iron chloride (FeCl_3_) (M.Wt = 162.20 g/mol, 97%) was (Sigma-Aldrich, Munich, Germany). The supplier of ethylene glycol (CH_2_OH)_2_ (M.Wt = 62.07 g/mol, 99%) was Alpha (India). The supplier of hydrated hydrazine (N_2_H_4_.H_2_O) (M.Wt = 50. 06 g/mol, 99%) was (Advent, Mumbai, India). The supplier of absolute ethanol (C_2_H_5_O) (M.Wt = 46. 07 g/mol, 99.8%) was (Piochem, 6th of October City, Egypt). Palladium chloride (PdCl_2_) (M.Wt = 177.326 g/mol), Potassium chloride (KCl) (M.Wt = 74.555 g/mol). N,N-dimethylethylenediamine (C_4_H_12_N_2_) (DMEN) (M.Wt = 88.144 g/mol), dimethyl sulfoxide (DMSO) (M.Wt = 78.13 g/mol) were purchased from (Sigma-Aldrich, Munich, Germany). Diethylnitrosamine (DEN) was acquired from Sigma-Aldrich Co. (St. Louis, MO, USA). Thioacetamide (TAA) was procured from (CAS No.: 62-55-5, Advent, Mumbai, India). DEN was reconstituted in 0.9% saline solution (Ghoneim International Group, Cairo, Egypt). TAA was dissolved in 0.9% saline solution prior to use. A standard spectrophotometric method was employed to measure the activities of aspartate aminotransferase (AST) and alanine aminotransferase (ALT) (Biodiagnostic Company, Alexandria, Egypt). All other chemicals were of the highest quality and standard. The enzyme-linked immunosorbent assay (ELISA) kits utilized for the quantification of Tumor Necrosis Factor-alpha (TNF-α) and Matrix Metalloproteinase-9 (MMP-9) were sourced from Elabscience, Houston, TX, USA, with catalog numbers E-EL-R3021 and E-EL-R2856, respectively.

### 2.2. Preparation of MNPs

The magnetite nanoparticles were synthesized utilizing the previously reported method [16] with some modifications. Particularly, 0.32 g of anhydrous FeCl_3_ was dissolved in 60 mL of ethylene glycol under vigorous stirring at room temperature to form a light-yellow solution. Then, to create a brown solution, a few drops of hydrated hydrazine (6 mL) were combined with the first solution. After the solution had turned brownish-brown, it was transfer to an 80-milliliter Teflon-lined stainless-steel autoclave (Ollital, Xiamen, China). The autoclave was then placed in the dryer (Daihan, Seoul, South Korea) for two days at 200 °C. Afterward, the resulting solution was left to cool down before being separated using the centrifuge (5000 rpm, 15 min, 20 °C, PrO-Research centrifuge, Bristol, UK), and the resulting powder was washed three times with distilled water subsequently by ethanol (Piochem, 6th of October City, Egypt), and dried for 2 days at 37 °C.

### 2.3. Preparation of Pd(DMEN)Cl_2_ Complex

The [Pd(DMEN)Cl_2_] complex was synthesized by heating PdCl_2_ (0.1773 g; 1.0 mmol) and KCl (0.1491 g; 2.0 mmol) in minimal water to 70 °C with continuous stirring [17]. The transparent K_2_[PdCl_4_] solution was cooled to 25 °C, filtered, and N,N-dimethylethylenediamine (0.0881 g; 1.0 mmol) was introduced to the stirred mixture. The solution was evaporated to a reduced volume of 20 mL under vacuum, resulting in the formation of an orange crystalline precipitate of [Pd(DMEN)Cl_2_] upon cooling. The precipitate was filtered and washed with water. An orange crystalline precipitate was obtained, resulting in a yield of 92%. The compound C_4_H_12_N_2_PdCl_2_ has a molecular weight of 265.47. The analytical composition is as follows: carbon (C) = 18.0%, hydrogen (H) = 4.7%, nitrogen (N) = 10.3%. The calculated composition is as follows: carbon (C) = 18.10%, hydrogen (H) = 4.56%, nitrogen (N) = 10.55%.

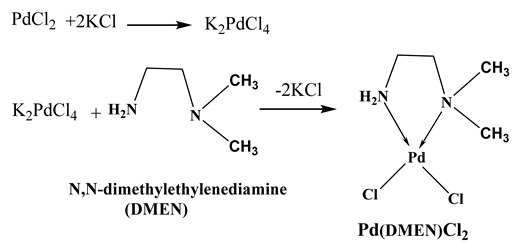



### 2.4. Loading of the Metal/Ligand Complex onto MNPs

Firstly, a solution of 0.1% of polyvinylpyrrolidone (Biobasic, Markham, ON, Canada) was dissolved in 100 mL of dimethyl sulfoxide (DMSO) as a dispersed agent. Subsequently, 100 mg of the magnetite nano-carriers were added to the solution above and left to stir normally. Following 30 min, 100 mg of the metal/ligand combination was dissolved in the same solution, and stirring was continued for an additional 30 min to make sure that the metal/ligand complex was fully dissolved and loaded onto the MPNs’ surface. The produced emulsions were then put in a shaker incubator (Thermoscientific, Waltham, MA, USA) set at 200 rpm for 2 h. Finally, the solvent was completely evaporated, and the metal/ligand complex-loaded magnetite nanoparticles were collected and kept in a fridge (Toshiba, 6th of October City, Egypt) for further investigations.

### 2.5. Characterization of the MNPs

The characteristics of the synthesized MNPs regarding crystallinity and amorphousness are examined using X-ray diffraction (XRD) analysis utilizing the BRUKER model (Discover-D8, Billerica, MA, USA). A vibrating sample magnetometer (VSM, Lake Shore Model 7410, Westerville, OH, USA) with an applied field of 31 kOe is employed to investigate the magnetic properties of the synthesized MNPs. Nitrogen adsorption–desorption tests were conducted at 77.35 K using a Nova Touch LX4 (Quantachrome, Boynton Beach, FL, USA) to determine the Brunauer–Emmett–Teller (BET) surface area and complete isotherm of MNPs. The BJH method was employed to ascertain the pore volume from the adsorption branch of the isotherm curve at P/Po = 0.995, assuming complete pore saturation. The size distribution and zeta potential of MNPs were analyzed using a Zetasizer Nano ZS instrument (Malvern Instruments, Great Malvern, UK) with a 633 nm laser, employing the light scattering method. The samples were solubilized in deionized water at 25 °C within a clear disposable zeta cell. The size distribution was measured at a distance of 5.50 mm from the Zeta cuvette wall. The zeta potential was measured 2 mm from the wall of the Zeta cuvette. The investigation of zeta potential was performed with the dispersion technology software (Version 7.13) from Malvern Instruments. The production of iron oxide as magnetic nanoparticles (MNPs) was confirmed by X-ray photoelectron spectroscopy (XPS). Measurements were conducted utilizing a SPECS spectrometer configuration (XR-50 source with monochromator-FOCUS 600, SPECS Surface Nano Analysis GmbH, Berlin, Germany) and Al Kα monochromatic radiation at an energy of 1486.7 eV. A hemispherical analyzer was employed for this purpose. Ultrahigh vacuum (UHV) was utilized at a pressure of 1109 mbar. The specimen was affixed to a sample holder via double-sided sticky carbon tape. The data was examined with Casa XPS processing and ULVAC-PHI MultiPak software (version 9.0.1, Osaka, Japan).

### 2.6. Characterization of the Metal/Ligand Complex and the Metal/Ligand Complex Loaded MNPs

#### 2.6.1. Molecular DFT Calculation

The equilibrium geometry of the ligand and complex at the B3LYP level of theory has been clarified using Density Functional Theory (DFT) calculations, where the Pd atoms are at lanl2dz and the C, H, N, and Cl atoms are at 6-311G++(dp) using the Gaussian 09 program [18].

#### 2.6.2. Thermal Analysis

Thermogravimetric Analysis (TGA) was conducted to examine the thermal properties of the metal/ligand complex Pd(DMEN)Cl_2_. Specifically, 10 mg of the compound was positioned in a platinum crucible under an air firing regime, with a temperature range of 25–700 °C and a firing rate of 5 °C min^−1^, utilizing the SDT Q600 V20.9Build 20 instrument (TA, New Castle, DE, USA). The outcomes were evaluated against aluminum oxide powder as a benchmark.

#### 2.6.3. FTIR Analysis

The FTIR spectrophotometer was used to investigate the metal/ligand complex [Pd(DMEN)Cl_2_] (C) and the metal/ligand complex loaded magnetite (C+Mag) in order to determine the chemical functional groups. Pure potassium bromide (KBr) powder (Sigma-Aldrich, St. Louis, MO, USA). was combined with the prepared ingredients to create pellets, which were subsequently examined using an FTIR equipment (model FT/IR-6100 type A, JASCO Corporation, Easton, PA, USA). The 400–4000 cm^−1^ wave number range was used to measure the spectra.

#### 2.6.4. TEM Analysis

Transmission electron microscopy (TEM) was employed to ascertain the shape and particle size of the metal/ligand complex [Pd(DMEN)Cl_2_] (C) and the magnetite-loaded metal/ligand complex (C+Mag) using a JEOL JEM-2100 electron microscope (JEOL, Tokyo, Japan). In total, 5 µL of the mixed metal/ligand complex-loaded magnetite were titrated onto a copper grid using Dimethyl Sulfoxide (DMSO). TEM images were acquired subsequent to the drying of the grid.

#### 2.6.5. Molecular Docking

Molecular docking investigations utilizing MOA2022 software. were performed to elucidate the probable binding mechanisms at the most active site of the methionine adenosyl-transferase receptor in liver cancer (PDB ID: 5A19) [18].

### 2.7. In Vitro Drug Release Behavior

The drug loading and encapsulation efficiency were determined using Equations (1) and (2). The amount of metal/ligand complex in the magnetite was determined by grinding the samples and then using gentle heat to extract the metal/ligand complex in methanol for 12 h. The solution was then filtered, diluted with PBS, and analyzed by UV spectrophotometry. The in vitro cumulative drug release of the metal/ligand complex [Pd(DMEN)Cl_2_] (C) from the magnetite (Mag) was conducted in phosphate-buffered saline (PBS) at 37 °C for distinctive intervals of time (1, 2, 4, 6, 24, 168, 336, 504, and 672 h) at two pH values, 7.4 and 4.5. The dialysis bag (the manufacturer, city, country) containing the metal/ligand complex-loaded magnetite was soaked in 100 mL of PBS. After each interval, 5 mL samples were withdrawn and replaced with fresh PBS to evaluate the metal/ligand complex [Pd(DMEN)Cl_2_] release. The metal/ligand complex [Pd(DMEN)Cl_2_] was measured using a UV spectrophotometer at a wavelength of 277 nm.

(1) $$\%\;Metal\;complex\;loading=\frac{Weight\;of\;metal\;complex\;in\;formulation}{Weigt\;of\;total\;formulation\;}\times \;100$$

(2) $$\%\;Encapsulation\;efficiency=\frac{Actual\;loading}{Theoretical\;loading\;}\times \;100$$

### 2.8. In Vivo Studies

#### 2.8.1. Experimental Animals

Ethical statement: Experiments using animals were conducted according to the guidelines of the Medical Research Ethics Committee (MREC) at NRC, approval number 190003. Fifty mature male Wister albino rats weighing 200 ± 20 g and aged 8–9 weeks were acquired from the NRC animal house in Cairo, Egypt. After two weeks of acclimatization, the rats were placed in groups of five in 56 × 38 × 22 cm polyacrylic cages. To keep the experimental animals stress-free, a 12 h light/dark cycle, a constant temperature of 25 ± 2 °C, and a humidity of 60 ± 5% were maintained. Rats were fed a typical chow diet and had unrestricted access to water. Five groups of ten rats each were created from the rats:

- Group I: Negative control rats: rats did not receive any intervention.
- Group II: Liver cancer-induced (DEN/TAA) positive control rats: Rats induced with hepatic cancer by receiving a single intraperitoneal (i.p.) injection of diethylnitrosamine [DEN, 200 mg/kg body weight, in 0.9% saline], and after two weeks, the rats were submitted to thioacetamide (TAA, 200 mg/kg body weight, i.p., in 0.9% saline) twice a week, according to Goto et al. [19].
- Group III: Liver cancer-induced (DEN/TAA) rats + MNP-treated rats: DEN+TAA-induced rats were treated with metal complex (10 mg/kg body weight; i.p.) once a week for 3 weeks
- Group IV: Liver cancer-induced (DEN/TAA) rats + metal/ligand complex-treated rats: DEN+TAA-induced rats were treated with metal complex/ligand (10 mg/kg body weight; i.p.) once a week for 3 weeks
- Group V: Liver cancer-induced (DEN/TAA) rats + metal/ligand complex-loaded magnetite: DEN+TAA-induced rats were treated with magnetite metal complex/ligand (10 mg/kg body weight; i.p.) once a week for 3 weeks.

#### 2.8.2. Macroscopic Examination of Liver Samples

Thiopental sodium (50 mg/kg, i.p., Sigma-Aldrich, Darmstadt, Germany) was used to anesthetize rats that were fasted overnight, with free access to water. Rats’ abdominal and thoracic chambers were exposed in order to analyze their liver tissue. After being individually cleaned in cold saline (Ghoneim International Group, Cairo, Egypt), the obtained livers were examined under a microscope to check for any pathological alterations.

#### 2.8.3. Collection of Blood and Liver Samples

Diethyl ether was used to anesthetize rats that had fasted overnight. Blood samples were taken and allowed to coagulate for 30 min at room temperature. The sera were separated by centrifugation at 2000 rpm for 10 min. Sera were aliquoted and kept at −80 °C. After collecting blood samples, rats were beheaded. The livers were dissected, washed, and preserved in 10% neutral buffered formalin for histopathology.

#### 2.8.4. Liver Function Markers

According to Reitman and Frankel [20], serum activities of aspartate transaminase (AST) and alanine transaminase (ALT) were estimated spectrophotometrically at 490 nm.

#### 2.8.5. Serum Inflammatory Markers

Serum TNF-α and MMP-9 levels were measured using ELISA technique following the manufacturer’s instructions (Elabscience, Houston, TX, USA). The data was expressed as *p*g/mL and ng/mL, respectively.

#### 2.8.6. Histopathological Analysis of Liver Tissues

Samples of liver were immediately preserved in a 10% buffered formalin saline solution (pH 7.4). Ethanol serial rising dilutions will be used to wash and dehydrate the liver samples. After that, they were placed in a hot-air oven set at 56 °C for 24 h while embedded in paraffin wax (ExxonMobil Chemical, Houston, TX, USA). The blocks of paraffin wax that had formed were divided into pieces that were 5 μm thick. Sections of liver were deparaffinized after being blotted onto glass slides. The liver sections were stained with hematoxylin and eosin (H&E) (Solarbio Science & Technology, Beijing, China) stain and subsequently seen under a light microscope. Morphological changes were photographed using a digital camera (DJI Technology Co, Shenzhen, China) that is linked to a computer system. Samples of liver tissue were examined for protein expression using immunohistochemical staining.

### 2.9. Statistical Analysis

The data were presented as mean ± standard error of the mean (SEM). The data were subjected to a basic one-way analysis of variance (ANOVA) with SPSS (2020, SPSS Statistics 27, Armonk, NY, USA), followed by Duncan’s multiple range tests. Statistical significance was defined as *p*-values < 0.05.

## 3. Results and Discussion

### 3.1. Characterization of MNPs

**Figure 1:**
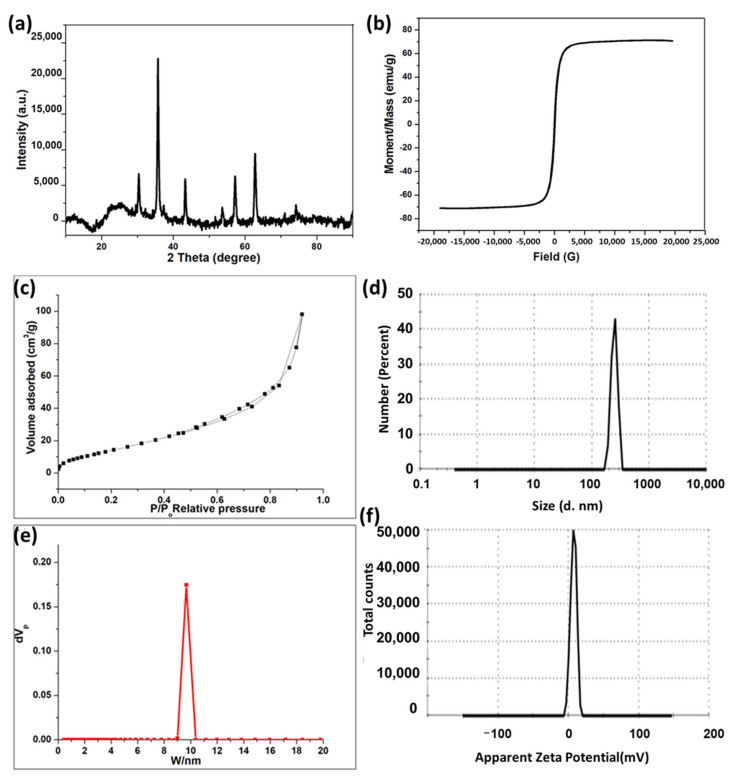
(**a**) XRD pattern of the MNPs. (**b**) Magnetic hysteresis enlarged partial (inset) curve of MNPs. (**c**) N_2_ adsorption–desorption isotherm of MNPs. (**d**) Cumulative pore volume of MNPs. (**e**) Particle size distribution curve of MNPs. (**f**) Zeta potential profile of MNPs.

The XRD pattern in Figure 1a illustrates that the MNPs were prepared in a crystalline form. The peaks resulting iron oxide were found to be matched with JCPDS card no. 19–629, they were detected at 2θ = 30.1, 35.8, 43.1, 53.8, 57.3, and 63.0° can be indexed to (220), (311), (400), (422), (511), and (440) [21,22]. Additionally, a vibrating sample magnetometer (VSM) was employed to characterize the hysteresis loops of MNPs in relation to the magnetic field at room temperature, as illustrated in Figure 1b. The magnetic property is one of the most crucial characteristics that facilitates medication delivery applications. The saturation magnetisation (Ms) of the magnetic nanoparticles (MNPs) was 71.273 emu/g, consistent with other studies on magnetite [23,24], while the coercivity (Hci) was 44.315 Oe. Consequently, the VSM test demonstrates the superparamagnetic properties of the synthesized MNPs.

The BET surface area, pore volume, and average pore radius of the synthesized MNPs are 63.75 m^2^/g, 0.1518 cm^3^/g, and 9.5 nm, respectively. Accordingly, it is suggested that the MNPs have large surface area and small pore radius, which increase the reactivity of the prepared material. Figure 1c illustrates the N_2_ adsorption–desorption isotherm of MNPs, which demonstrates hysteresis behavior, indicating the presence of open pores within the particles, as per the IUPAC categorization [25]. Figure 1d represented the pore size distribution analysis of MNPs (PDI: 0.15), which illustrates the presence of nanopores through the prepared magnetite. The particle size distribution and zeta potential of the prepared MNPs were performed by using a Zetasizer instrument. It is important to know the charge of the drug carriers as it plays a critical role in the delivering of bioactive molecules. The particle size distribution of MPNs is shown in Figure 1e. It exhibits a very sharp peak, with an average particle diameter of 246.8 nm in 100% (very narrow distribution). Figure 1f illustrates the zeta potential of MNPs which possessed a value of 7.32 mV. It is worth noting that the agglomeration of the prepared material might affect the size of MNPs that appears here in this analysis and differs from TEM analysis [26,27].

**Figure 2:**
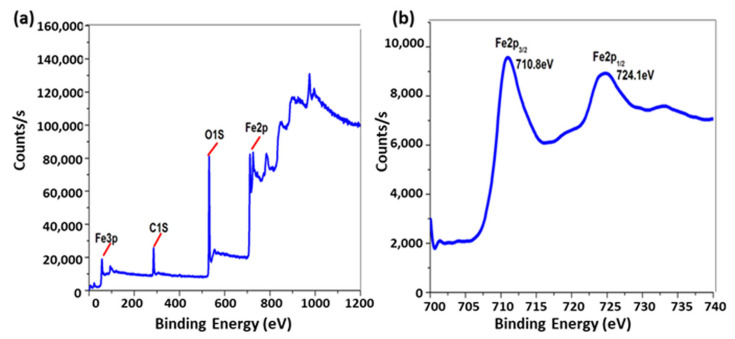
(**a**) XPS survey spectra and (**b**) high-resolution Fe 2p of Fe_3_O_4_.

XPS analysis is employed to identify the oxide type of the obtained MNPs. Figure 2a illustrates the whole scanned XPS spectra of MNPs within the 0–1200 eV range, revealing no additional peaks aside from Fe 2p, O 1s, and C 1s peaks. The C peak arose from surface contamination in the analyzed samples. Figure 2b illustrates the high-resolution Fe 2p spectra of MNPs, displaying two peaks: Fe 2p3/2 and Fe 2p1/2. The MNP spectra exhibit two peaks at binding energies of 710.8 eV and 724.1 eV for Fe 2p3/2 and Fe 2p1/2, respectively, consistent with the Fe_3_O_4_ values documented in the literature [28,29,30,31]. The lack of a shake-up satellite peak at around 719.3 eV, which is indicative of Fe_2_O_3_ [31,32,33], which serves as evidence for the presence of the Fe_3_O_4_ form. The inclusion of Fe^2+^ and Fe^3+^ ions in MNPs led to a little chemical shift, resulting in the broadening of the spin–orbit split Fe 2p peaks [29,34].

### 3.2. Characterization of Metal/Ligand Complex Pd(DMEN)Cl_2_ Acid-Base Equilibria of [Pd(DMEN)(H_2_O)_2_]^2+^ Complex

**Figure 3:**
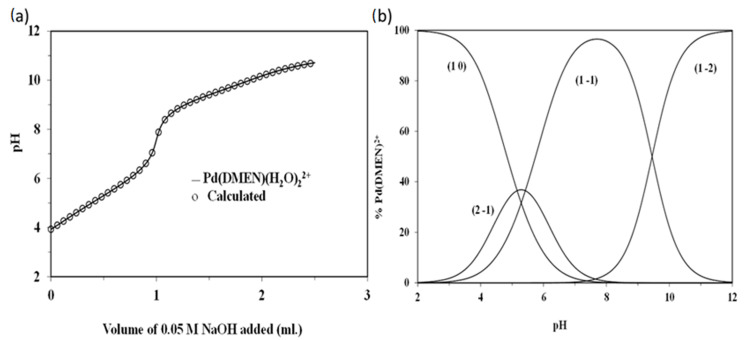
(**a**) Potentiometric titration curve of 0.05 mmoles of [Pd(DMEN)(H_2_O)_2_]^2+^, (**b**) Concentration distribution of various species as a function of pH in the Pd(DMEN)-OH system (at a concentration of 1.25 mmole/liter for [Pd(DMEN)(H_2_O)_2_]^2+^).

Coordinated water molecules frequently exhibit greater acidity than bulk water molecules. [Pd(DMEN)(H_2_O)_2_]^2+^ is susceptible to hydrolysis. The acid-base chemistry was ascertained by fitting the potentiometric data to several acid-base models, as seen in Figure 3a. Table 1 demonstrates that the optimal model remained consistent across three species (1-1, 1-2, and 2-1). The initial two, 1-1 and 1-2, originate from the deprotonation of the two coordinated water molecules, as delineated in Equations (3) and (4). Per Equation (3), the third species, 2-1, is the dimeric form of the monohydroxo complex (1-1) associated with the [Pd(DMEN)(H_2_O)_2_]^2+^ species (1 0).

pKa1 [Pd(DMEN)(H_2_O)_2_]^2+^ ⇌ [Pd(DMEN)(H_2_O)(OH)]^+^+ H^+^ 1 0                                                                                            1-1(3)

pKa2 [Pd(DMEN)(H_2_O)(OH)]^+^ ⇌ [Pd(DMEN)(OH)_2_] + H^+^ 1 0                                                                                            1-1(4)

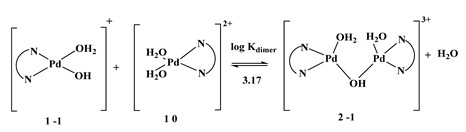

The pKa1 and pKa2 values for [Pd(DMEN)(H_2_O)_2_]^2+^ are 5.29 and 9.45, respectively. The equilibrium constant for dimerisation (3) can be determined using the following relationship:

log Kdimer = log β2-1 − logβ1-1 = −2.12 − (−5.29) = 3.17

[Pd(DMEN)(H_2_O)_2_]^2+^ and its hydrolyzed species concentration distribution plot is displayed in Figure 3b. The monohydroxo species, 1-1, has a concentration that rises as pH rises until it reaches its maximum concentration of 96.5% at pH 7.8. The maximal concentration of the dimeric species, 2-1, is approximately 36.6% at pH 5.2. In the pH range of approximately 5.4 to 9.6, the monohydroxo species (1-1) is the predominant species, indicating its presence under physiological conditions. As the pH rises further, the dihydroxo species (1-2), the primary species above pH ~ 9.6, also rise.

#### 3.2.1. Molecular DFT Calculation of Ligands (DMEN)

**Figure 4:**
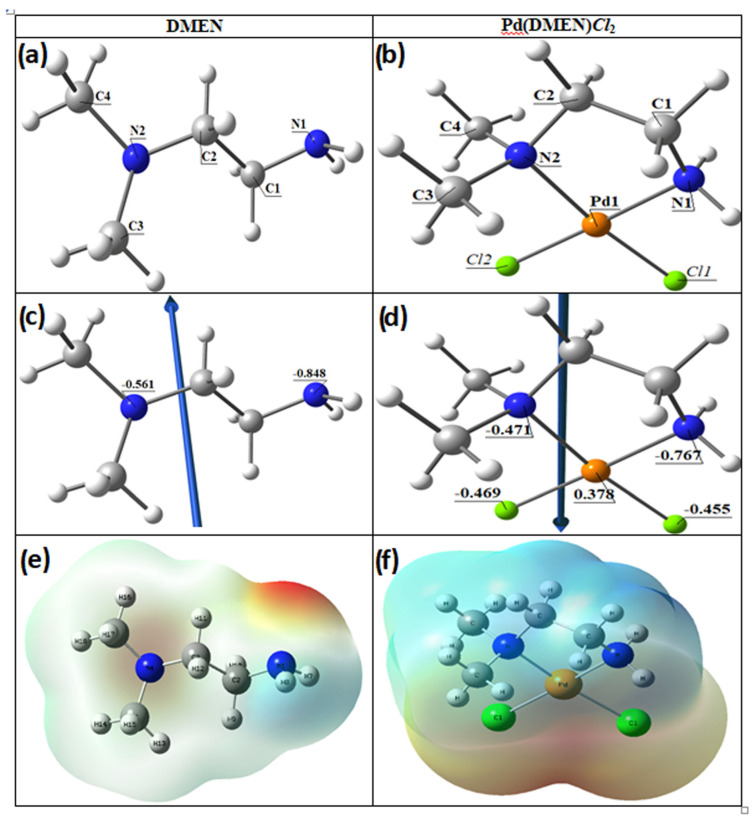
The optimized structures of (**a**) the ligand DMEN, (**b**) the complex, [Pd(DMEN)Cl_2_], the vector of the dipole moment of (**c**) the ligand DMEN, (**d**) the complex, [Pd(DMEN)Cl_2_], the natural charges on atoms, and molecular electrostatic potential (MEP) surface of (**e**) the ligand DMEN and (**f**) the complex, [Pd(DMEN)Cl_2_].

The Ligand (DMEN) optimized structure is the lowest energy arrangement, as seen in Figure 4. N1 (−0.848) and N2 (−0.561) are the most negatively charged active sites of DMEN, according to the Natural Bond Orbital Analysis (NBO). N2 forms five-membered rings, and the metal ions favor bidentate coordination over N1. The color of the ligand’s molecular electrostatic potential (MEP) surface varies from red, which is more negative, to blue, which is more positive.

#### 3.2.2. Molecular DFT Calculation of [Pd(DMEN)Cl_2_] Complex

Figure 4 illustrates the optimized lowest energy configurations of the compound [Pd(DMEN)Cl_2_]. The palladium atom exhibits four-coordinate square planar geometry, with atoms N1, N2, Cl2, and Cl1 nearly coplanar, deviating by −0.479° in the complex [Pd(DMEN)Cl_2_]. The bond angles in the square planar configuration approximate 90°, varying from 84.37° to 95.53°, with angles N1-Pd-Cl2 and N2-Pd-Cl1 nearing linearity, as shown in Table 2. The distance between N1 and N2 diminished upon complex formation from 3.769 Å in the free ligand to 2.864 Å in the complex [Pd(DMEN)Cl_2_], demonstrating robust coordination between the palladium metal and nitrogen atoms.

**Figure 5:**
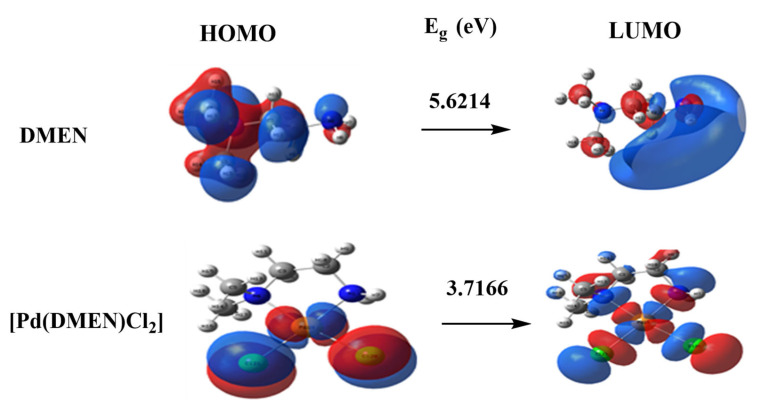
HOMO and LUMO charge density maps of DMEN and [Pd(DMEN)*Cl*_2_] complex.

The dipole moment computes are represented in Table 3 demonstrated the highest occupied molecular orbital (HOMO) energies, lowest unoccupied molecular orbital (LUMO) energies, and total energy for the ligands and complexes. The reduced total energy of the complexes relative to the free ligand indicates that the complexes exhibit greater stability than the free ligands. The metal ions interact with the ligand, resulting in reduced energy gaps (Eg = ELUMO − EHOMO) in the complexes compared to the ligand (Table 3). The charge transfer interactions in complex formation are elucidated by the reduction in Eg in the complex relative to that of the ligand (Figure 5). Figure 5 illustrates the charge density maps of the HOMO and LUMO for the DMEN and [Pd(DMEN)Cl_2_] complex.

#### 3.2.3. Reactivity Studies

A multitudeof reactivity descriptors has been developed to elucidate various facets of reactivity associated with chemical processes. These encompass ionization potential (I), electron affinity (A), electronegativity (χ), chemical potential (μ), hardness (η), softness (S), and electrophilicity index (ω). All descriptors are derived from the energies of the HOMO and LUMO (Table 2).

#### 3.2.4. Molecular Docking

Molecular docking investigations were performed using MOA2022 software to elucidate the putative binding processes of the main active site of the methionine adenosyl-transferases receptor in liver cancer (PDB ID: 5A19) [19,35]. The binding free energies of the ligand DMEN and the complex [Pd(DMEN)Cl_2_] with the receptor of methionine adenosyl-transferases in liver cancer (PDB ID: 5A19) are −7.1 and −10.1 kcal/mol, respectively, as indicated in Table 4. A lower binding energy indicates a more robust connection.

DMEN ˂ [Pd(DMEN)Cl_2_]

**Figure 6:**
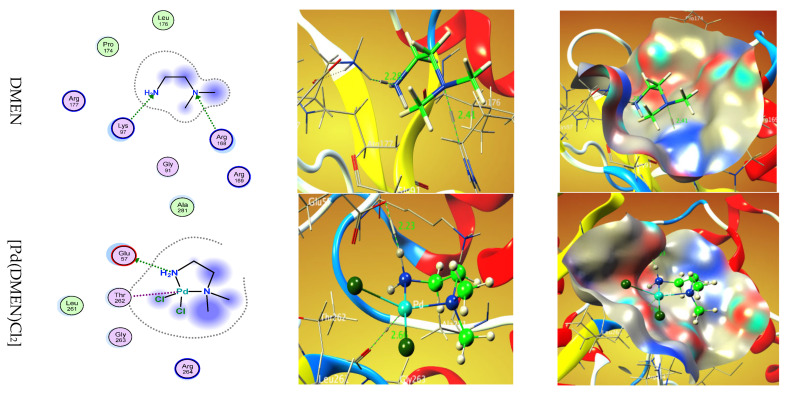
Two-dimensional and 3D plots of the interaction between DMEN and [Pd(DMEN)Cl_2_] with the active site of the receptor of liver cancer protein (PDB ID: 5A19). Hydrophobic interactions with amino acid residues are shown with dotted curves.

Figure 6 displays the 2D and 3D representations of the interaction between DMEN and [Pd(DMEN)Cl_2_] with the active site of the liver cancer protein receptor (PDB ID: 5A19).

### 3.3. Thermogravimetric Analysis

**Figure 7:**
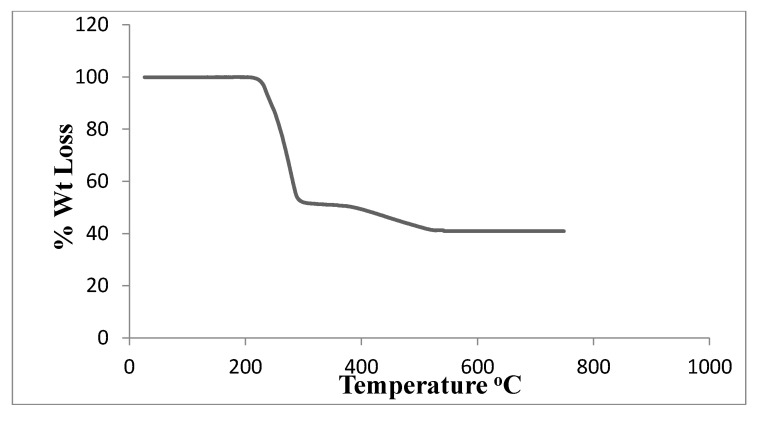
TGA of [Pd(DMEN)Cl_2_] complex.

The complex’s thermogravimetric analysis was performed at temperatures ranging from ambient temperature to 700 °C. Table 5 displays the thermal impacts and percentage mass loss related to the solid complex’s changes upon heating. Figure 7 depicts two stages in the TGA curve. The ligand (C_4_H_12_N_2_) + one Cl atom is lost between 220 and 320 °C in the first step, and the other Cl atom is lost between 320 and 570 °C in the second step. Pd, which has a theoretical value of 40.09%, is represented by the residue of 40.01 percent. It is easier to lose the first Cl^−^ from neutral PdCl_2_ than the breaking of the second Cl^−^ from positive PdCl^+^, which needs more energy to break. The thermal analysis curve shows that the percentage loss in the first step is 47.07% which closely corresponds to the theoretical percentage of 46.56% for the whole ligand and one Cl atom. The Pd^2+^ atom is strongly attached to two Cl^−^; the first Cl^−^ is more easily lost from neutral PdCl_2_ at lower temperature than the second one, which requires more energy to detach from the positively charged PdCl^+^ fragment.

### 3.4. Metal/Ligand Complex Pd(DMEN)Cl2 Loaded Magnetite

#### 3.4.1. FTIR Analysis

**Figure 8:**
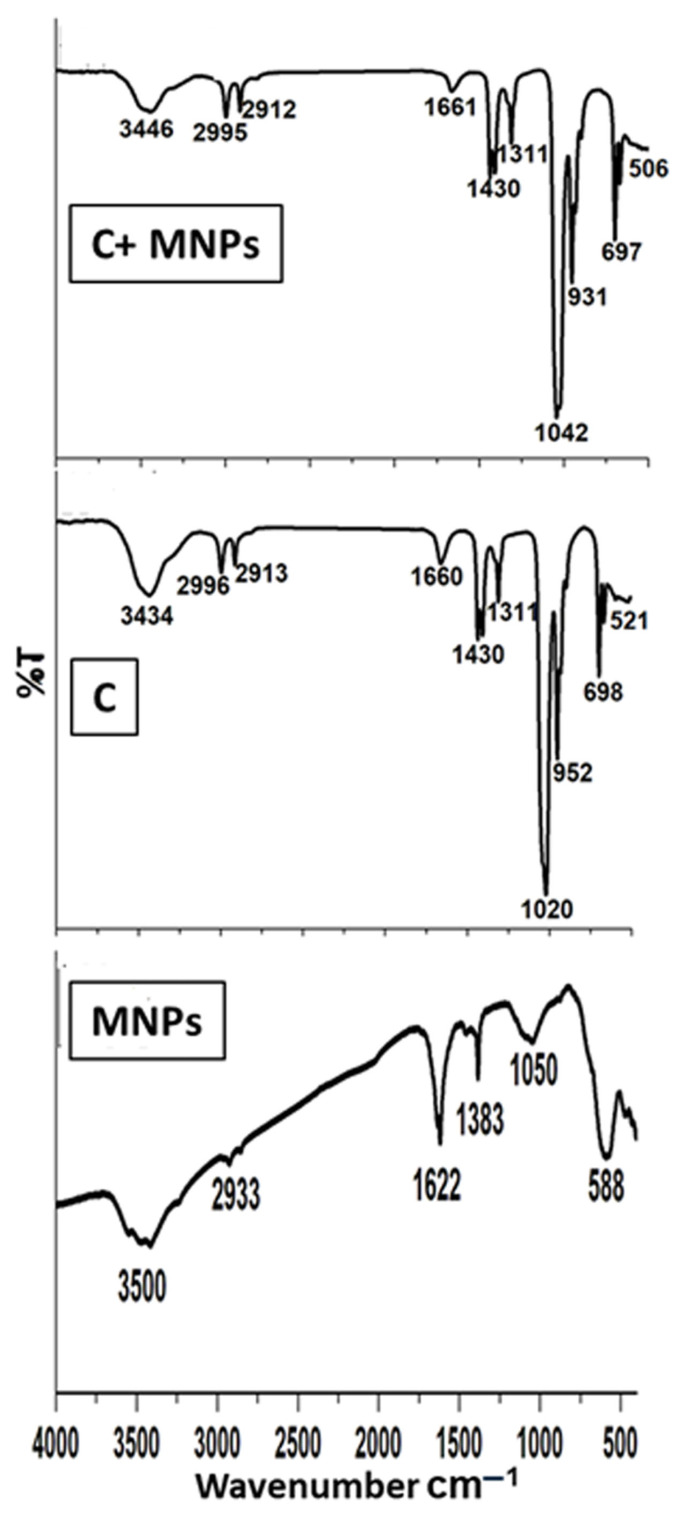
FTIR spectra of the MNPs, metal/ligand complex [Pd(DMEN)Cl_2_], and metal/ligand complex-loaded magnetite.

Figure 8 illustrates the FTIR spectrum of the synthesized magnetite MNPs, the metal/ligand complex [Pd(DMEN)Cl_2_] (C), and the metal/ligand complex [Pd(DMEN)Cl_2_] in conjunction with magnetite (C+MNPs). Magnetite exhibited a pronounced wide band at 3500 cm^−1^ and a prominent small band at 1622 cm^−1^, corresponding to the stretching and bending of O–H, respectively. The sample MNPs exhibited a pronounced broad band at 3500 cm^−1^ and a distinct sharp band at 1622 cm^−1^, corresponding to the stretching vibrations of O–H and the bending of O–H, respectively [36,37]. Furthermore, the C–H stretching vibrations and bending bands seen at 2933 and 1383 cm^−1^ are caused by the alcoholic group (ethylene glycol) used as a starting material. Alcoholic derivatives’ C–O stretching vibrations are responsible for the band at 1050 cm [38]. A FeO bond vibration at 588 cm^−1^ was recorded, confirming the production of iron oxide [39,40]. A sample of the metal complex (C) exhibited the distinctive bands corresponding to the functional groups of the complex [Pd(DMEN)Cl_2_]. A large band at 3434 cm^−1^ corresponds to the N–H stretching vibrations of the main amine [41]. The bands at 2996 and 2913 cm^−1^ correspond to C–H stretching vibrations for CH_2_ and CH_3_ groups [42]. A band for bending N–H (primary amine) is observed around 1660 cm^−1^. The bending C–H group was observed at 1430 cm^−1^ [43,44]. The band at 1311 cm^−1^ is attributed to the bending of the methyl group (CH_3_) [41,42]. The band at 1020 cm^−1^ is ascribed to the stretching of C–N (aliphatic amine) [41,42,43,44]. A band at 952 cm^−1^ corresponds to bending C–H (disubstituted). The band at 698 cm^−1^ is ascribed to the wagging motion of the N–H bond in primary amines. The band at 521 cm^−1^ is attributed to the stretching vibrations of Pd–N [44,45,46]. The spectrum of the metal/ligand complex [Pd(DMEN)Cl_2_] on magnetite indicated that certain bands shifted as a result of the interaction between the magnetic nanoparticles and the complex, specifically the bands at 3446, 1042, and 506 cm^−1^.

#### 3.4.2. TEM Analysis

**Figure 9:**
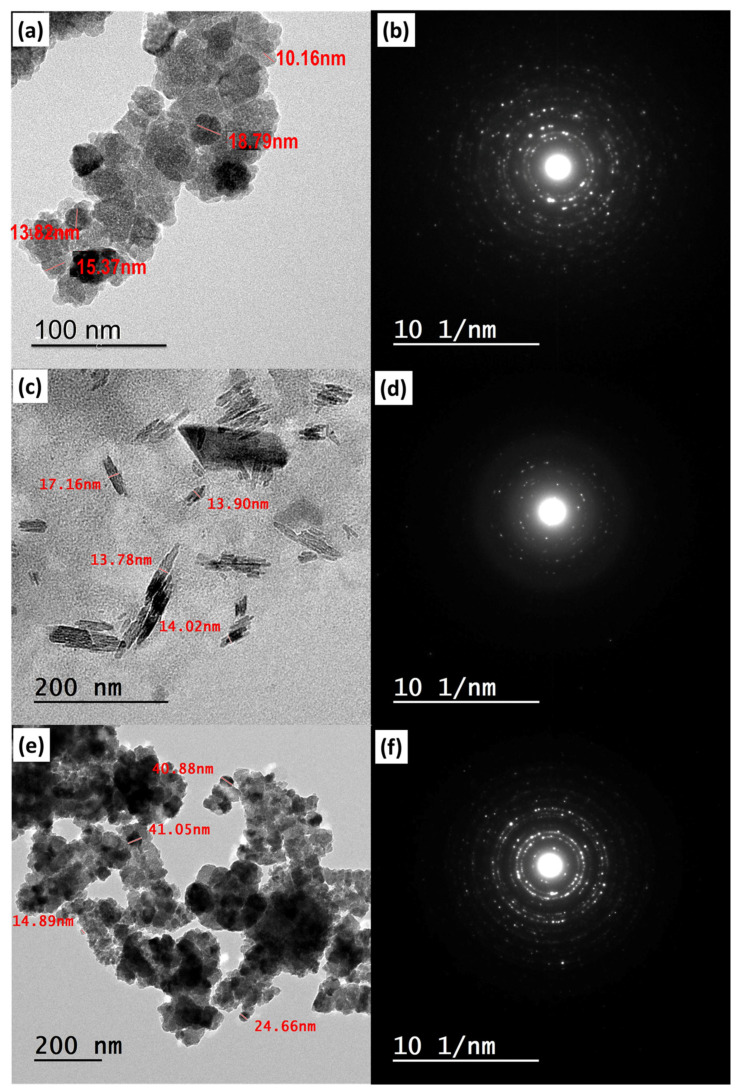
TEM images of (**a**) the MNPs, (**b**) their diffraction pattern, (**c**) metal/ligand complex [Pd(DMEN)Cl_2_], (**d**) its diffraction pattern, (**e**) metal/ligand complex-loaded magnetite and its diffraction pattern (**f**).

The morphology of the MNPs, the metal/ligand complex [Pd(DMEN)Cl_2_], and the magnetite loaded with the metal/ligand complex [Pd(DMEN)Cl_2_] was shown in Figure 9. It was confirmed that the MNPs are present in nanosize (Figure 9a), with an average diameter below 20 nm. Figure 9b illustrates the MNPs diffraction pattern, which confirms the formation of magnetite in crystalline form. Figure 9c displayed the shape of the complex [Pd(DMEN)Cl_2_], which looked like rods and measured between 13 and 18 nm in size. Figure 9d displayed the diffraction pattern of the complex [Pd(DMEN)Cl_2_], which examined its crystal structure. Figure 9e displayed the MNPs mixed with the metal/ligand complex [Pd(DMEN)Cl_2_], showing how the complex altered the shape of the MNPs. The metal ions or complexes can change the zeta potential of nanoparticles, which can either encourage or prevent them from clumping together based on the balance of charges. The modification resulted in an increase in the nanosize to 41 nm, highlighting the dark areas that represent the MNPs and the bright areas that signify the complex [47,48,49,50]. Metal complexes alter Mag surface chemistry and zeta potential, directly affecting agglomeration through modified electrostatic interactions and steric properties that can either stabilize or destabilize particle dispersions. The stabilization effect depends on the metal complex’s acidity, solution pH, and the formation of low-melting compounds that influence particle crystallinity and aggregation behavior [48,49]. Figure 9f illustrates the diffraction pattern of the metal/ligand complex [Pd(DMEN)Cl_2_]-loaded MNPs that confirms the formation of a crystalline form.

### 3.5. In Vitro Drug Release Behavior

**Figure 10:**
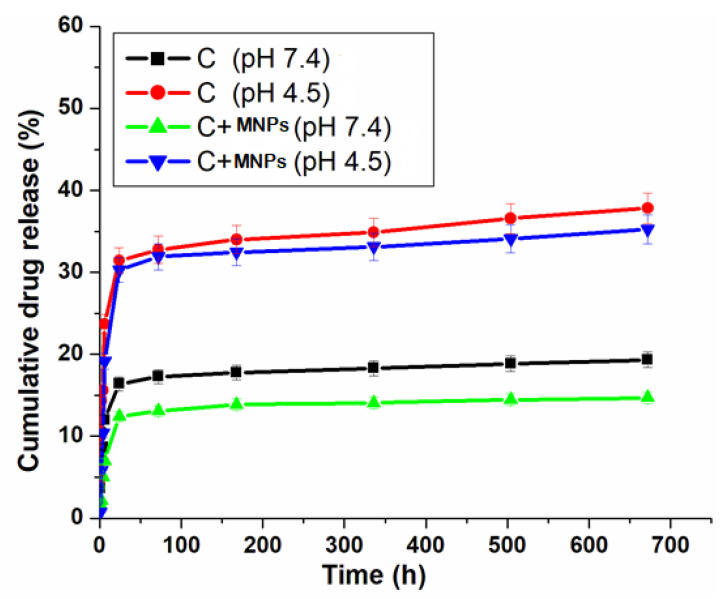
Cumulative drug release (%) of metal/ligand complex [Pd(DMEN)Cl_2_] measured in PBS at different 2 pH 7.4 and 4.5.

The loading efficiency was found to be approximately 50%, and the encapsulation efficiency was around 70–85%, though actual values will be lower due to incomplete surface adsorption and solvent evaporation losses. Not having steps to separate unbound drugs and using a basic physical adsorption method instead of chemical bonding greatly lowers both efficiencies from their highest possible levels. The cumulative drug release profiles of complex [Pd(DMEN)Cl_2_] (C) in the presence and absence of MNPs at two different pH levels, 7.4 and 4.5, are represented in Figure 10. The pH of normal cells is 7.4, and the pH of liver cancer cells is acidic, 4.5. This difference in pH helps in creating a drug delivery system that could be controlled to release their drug in the target sites (cancer cells) and prevent their release in the bloodstream to avoid the side effects of the anticancer drugs [51]. Figure 10 shows that the release of the drug (complex) depends on pH, with more drug being released at pH 4.5 than at pH 7.4, which matches earlier research. The release of the complex with MNPs helps the drug to be released steadily, which stops it from spreading out before reaches the tumor cells [48].

At pH 4.5, the metal complex releases at an elevated rate, mimicking the acidic microenvironment of liver cancer cells. This pH-sensitive system helps deliver medicine directly to the tumor, ensuring the drug is released where it is needed most. The improved release at this lower pH is likely due to the drug complex gaining protons, which may help it dissolve better and be released from the carrier. At a pH of 7.4, which is typical for the body, the drug release is significantly lower. The drug release significantly decreases at pH 7.4, which is representative of normal physiological conditions. This feature is important for lowering the overall exposure to the drug in the body and reducing side effects, as the drug mainly stays in the delivery path until it reaches the cancerous tissue. The presence of MNPs improves the controlled release mechanism. It has a prolonged release profile, inhibiting early drug distribution in the bloodstream. This feature ensures the confinement of the medicine to the tumor location, thereby enhancing therapeutic efficacy and minimizing toxicity [52]. The way the complex is released based on pH shows that we can create better ways to deliver anticancer that take advantage of the unique acidic environment of tumors. This method enhances drug accessibility at the target location while reducing the negative effects linked to anticancer treatments. 

### 3.6. In Vivo Studies

#### 3.6.1. Macroscopic Examinations of Liver Samples

**Figure 11:**
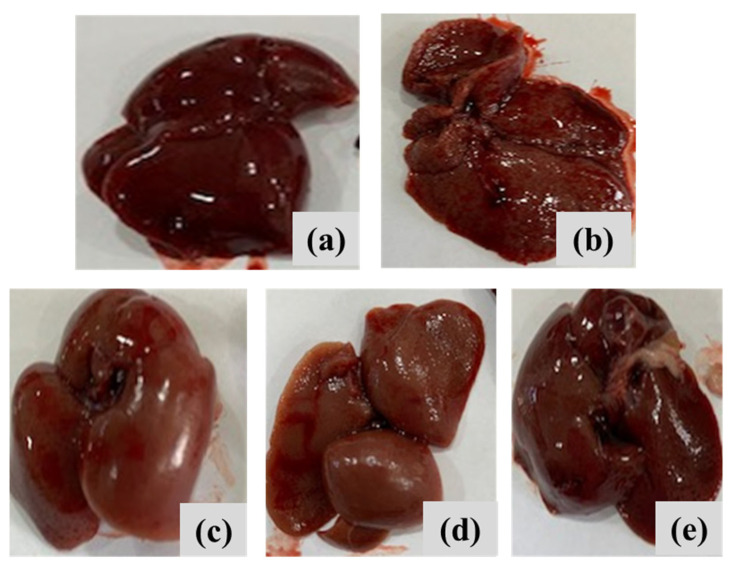
Macroscopic examination of liver samples of different experimental groups of (**a**) negative control rats, (**b**) liver cancer-induced DEN/TAA rats, (**c**) liver cancer-induced DEN/TAA rats treated with MNPs, (**d**) liver cancer-induced DEN/TAA rats treated with complex, and (**e**) liver cancer-induced DEN/TAA rats treated with complex loaded MNPs.

Figure 11 depicts the overall appearance of liver tissues across all groups. The findings indicated that the negative control rats had smooth liver surfaces with uniform lobes (Figure 11a). The positive control DEN/TAA-induced rats, afflicted with liver cancer, exhibited a nodular liver surface characterized by numerous big and tiny masses. Rats with liver cancer treated with MNPs, a complex, or complex-loaded MNPs exhibited an almost normal appearance, characterized by a smooth liver surface equivalent to that of the control liver samples (Figure 11c–e). This study sought to investigate the therapeutic effects of MNPs compounds on hepatocarcinogenesis caused by a DEN/TAA protocol in rats. The experimental DEN model serves as a preclinical model for hepatocellular carcinoma, displaying numerous phenotypic traits pertinent to liver cancer [53].

#### 3.6.2. Effects of Treatment of DEN/TAA Rats with Metal/Ligand Complex [Pd(DMEN)Cl_2_], on Hepatic Activities

Compared to the negative control rats, administrating rats with DEN and TAA to induce liver cancer led to a significant increase in serum AST and ALT activities (53.26% and 24.88%, respectively). On the other side, treatment of liver cancer-induced rats with MNPs resulted in a significant decrease in AST and ALT activities by −33.16 and −19.83%, respectively for the MNPs, −33.97 and −17.69%, respectively for the complex compound, and −34.75 and 21%, respectively for the complex loaded MNPs. The present study indicated that liver-cancer DEN/TAA rats exhibited significant up-regulation of the liver biomarkers (ALT and AST). Giving DEN usually leads to gradual liver inflammation and scarring, which is then followed by the development of tumor lumps. DEN, a common substance that can cause liver cancer, starts the cancer process by causing breaks in DNA and creating harmful reactive compounds that lead to liver cancer. Many studies on liver damage caused by DEN showed increased levels of blood markers that indicate liver cell damage and problems with liver function. The liver dysfunction in DEN/TAA rats may be attributed to TAA’s capacity to alter the liver’s biochemical state, potentially resulting in significant hepatic damage. Furthermore, TAA markedly elevated hepatic enzymatic activity, resulting in an enhanced release of liver enzymes into the bloodstream [54,55]. These results indicated that treatment of DEN/TAA rats with MNPs, complex, or complex loaded MNPs mitigated the liver dysfunction in different treated groups. However, complex-loaded MNPs exhibited the best therapeutic potential through demonstrating the highest improvement of liver function indices ALT and AST, as indicated in Table 6.

#### 3.6.3. Effects of Treatment of DEN/TAA Rats with Complex Compounds on the Inflammatory Markers TNF-α and MMP-9

TNF-α plays a dual role in neoplastic progression; it initially induces necrosis, which promotes the proliferation of fibroblasts and other tumor-associated cells, subsequently disrupting the cancer vasculature. TNF-α enhances the synthesis of genotoxic compounds and stimulates the NF-kB-dependent anti-apoptotic factors; TNF-α is crucial in inflammatory responses and the initiation of apoptosis. MMP-9 is a substrate-specific gelatinase that contributes to the destruction of the extracellular matrix (ECM). Increased MMP-9 levels are associated with invasion and metastases in many malignancies [56]. MMP-9 is a metalloproteinase that plays a crucial role in angiogenesis, metastasis, and oncogenesis. Consequently, MMP-9 inhibitors have emerged as a prospective target for the development of anticancer pharmaceuticals [57,58].

The DEN-induced onset of hepatic malignancy affected Kupffer cells by stimulating the production of several inflammatory mediators, including TNF-α and MMP-9. The DEN/TAA rat model of liver cancer demonstrated a notable increase in TNF-α and MMP-9 levels (51.92% and 119.79%, respectively) in comparison to the negative control rats as demonstrated in Table 7. Our findings align with those of Abdel-Hamid et al. [59], Kurma et al. [60], and Singh et al. [61].

Regarding the impact of treatment on serum TNF-α and MMP-9 levels, treatment of DEN/TAA-induced rats with MNPs, or complex, or complex-loaded MNPs triggered significant reductions in serum TNF-α level by −16.12, −18.39, and −26.1%, respectively, as compared to DEN/TAA rats. On the other hand, serum MMP-9 level demonstrated non-significant change for DEN/TAA rats treated with MNPs (−2.36%), significant increase for DEN/TAA rats treated with complex (+108.59%), and significant decrease for DEN/TAA rats treated with complex-loaded MNPs (−40.87%), as compared to DEN/TAA rats. From our results, we could deduce that treatment of DEN/TAA rats with complex-loaded MNPs showed the highest therapeutic potential for liver cancer by stimulating synergistic decrement of serum levels of both TNF-α and MMP-9 (−26.09 and −40.87%, respectively) as compared to the DEN/TAA rats, as indicated in Table 7. The unexpected increase in MMP-9 in the treated DEN/TAA rats with a complex might be ascribed to the potential existence of pro-metastatic risks, which contradicts the therapeutic goal of treatment with the complex alone. Hence, complex-loaded MNPs could be regarded as a potent MMP-9 inhibitor, with anti-inflammatory activities, which could be an effective treatment against liver cancer.

#### 3.6.4. Histopathological Analysis of Liver Tissues

**Figure 12:**
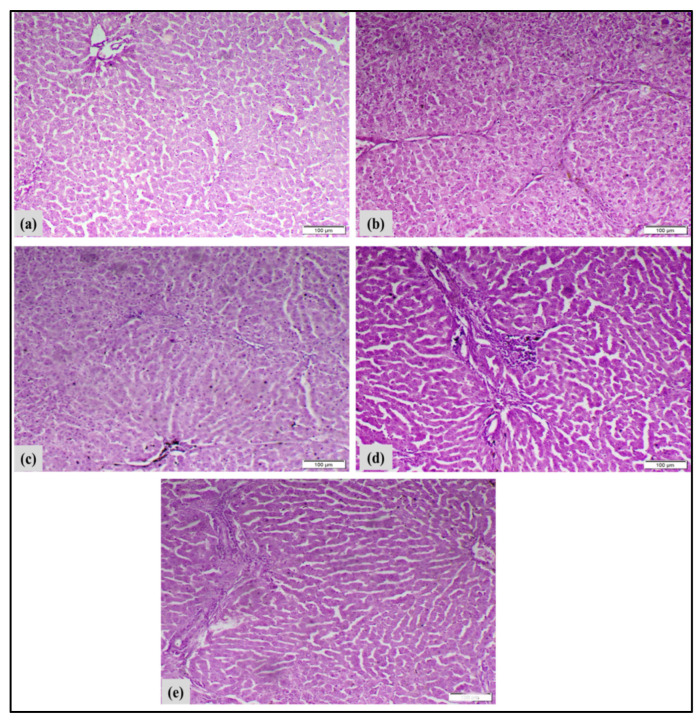
Illustrates the impact of metal/ligand complex [Pd(DMEN)Cl_2_] treatment on the development of liver cancer in various experimental groups. (**a**) Negative-control rats: Microscopic liver sections showed the normal histological structure of hepatic tissue (H&E, scale bar 100 µm). (**b**) In rats with liver cancer caused by DEN/TAA, microscopic liver sections showed significant damage to liver cells and cell death, along with a noticeable increase in fibroblasts and inflammation from immune cells. A pseudocopulation was found (H&E, scale bar 100 µm). (**c**) Liver cancer + MNP-treated rats, looking at liver samples under a microscope revealed some damage to liver cells, inflammation from immune cells, and an increase in certain types of cells that help with tissue repair (H&E, scale bar 100 µm). (**d**) Liver cancer + complex-treated rats, looking at liver samples under a microscope revealed some damage to liver cells, inflammation from immune cells, and an increase in certain types of cells that help with tissue repair (H&E, scale bar 100 µm). (**e**) Liver cancer + complex-loaded MNP-treated rats: Under a microscope, liver samples showed slight damage to liver cells and cell death, a moderate number of immune cells, and an increase in fibroblasts and cholangiocytes (H&E, scale bar 100 µm).

The liver tissues were stained with hematoxylin and eosin (H&E) to illustrate histological alterations, as depicted in Figure 12. When looking at the liver tissues from the control rats under a microscope, they showed normal cell structure, healthy cytoplasm, and a clear nucleus, with noticeable layers of liver cells (Figure 12a). Liver cancer-induced DEN/TAA rats exhibited irregular hepatocyte structures. Fibrous septa extending from the central vein to the portal area partitioned the liver tissue, signifying significant inflammation (Figure 12b). Rats with liver cancer that were treated with MNPs showed mild damage to liver cells, cell death, a moderate number of inflammatory cells, and an increase in certain types of cells that help with tissue repair (Figure 12c). Treated liver cancer-induced rats with complex demonstrated moderate hepatocytic degeneration and necrosis, moderate mononuclear inflammatory cell infiltration, and proliferation of fibroblasts and cholangiocytes (Figure 12d). Treated liver cancer-induced rats with complex loaded MNPs showed slight liver cell damage and death, moderate inflammation from immune cells, and growth of fibroblasts and cholangiocytes (Figure 12e). The histopathological results showed that complex-loaded MNP treatment had the best therapeutic effect compared to the complex and MNPs separately, through mitigating DEN/TAA-induced structural alterations in the hepatic tissue.

Macroscopically, livers of DEN/TAA induced rats exhibited abnormal gross appearance and notable hepatomegaly. Histopathological examinations of the liver tissues validated the biochemical results. Histopathological analysis of DEN/TAA-induced liver tissue revealed hepatocellular injury leading to the development of hepatocellular carcinoma (HCC). This finding aligns with Santos et al. [62], who indicated that DEN-induced HCC exhibits histological and genetic similarities to human tumors. Furthermore, Ojeaburu and Oriakhi [63] have demonstrated the ability of TAA to produce hepatic injury. Conversely, the administration of the metal/ligand complex [Pd(DMEN)Cl_2_] to induced rats decreased the elevated ALT and AST activities, and the hepatocytes in liver sections of treated rats exhibited nearly normal organization and architecture by inhibiting the leakage of hepatic enzymes and mitigating hepatocyte inflammation. Likewise, the administration of the metal/ligand complex [Pd(DMEN)Cl_2_] compounds to induced rats mitigated the macroscopic changes to varying extents. Treatment of rats with liver cancer using metal/ligand complex [Pd(DMEN)Cl_2_] compounds resulted in considerable enhancement of liver function and architecture, indicating the therapeutic efficacy of these compounds against DEN/TAA-induced liver cancer.

## 4. Conclusions

In this study, superparamagnetic, crystalline, and porous magnetite MNPs loaded with [Pd(DMEN)Cl_2_] complexes were successfully developed for target drug delivery. The [Pd(DMEN)Cl_2_] complex had a square planar geometry and attached well to methionine adenyltransferase receptors, performing better than the free ligands. FTIR and TEM tests indicated that the [Pd(DMEN)Cl_2_] complex was successfully attached to MNPs, forming a stable system. Drug release studies indicated that more of the drug was released in an acidic environment—similar to what is found in tumors—while less was released under normal pH conditions. The DEN/TAA-induced rats with liver cancer that received the complex-loaded MNPs showed improved anticancer potential, effectively exerting anti-inflammatory activity against DEN/TAA-induced hepatocarcinogenesis by reducing TNF-α levels and inhibiting MMP-9 levels by −26.09 and −40.87%, respectively. Therefore, complex-loaded MNPS could be regarded as a potential nanoformulated inhibitor of MMP-9 with potent anticancer activity. This study may contribute to the development of novel, potent, and selective MMP-9 inhibitors for liver cancer treatment in the future.

## 5. Study Limitations

While our results present valuable insights, further validation using both in vitro (i.e., primary cancer cells) and in vivo systems of liver cancer, in addition to including different treatment groups of nanoformulated MNPs loaded with clinical chemodrugs, would be important to validate the therapeutic anticancer potency and safety of the developed compounds and to provide more applicable strategies. Further research is required to conduct biodistribution assays and survival curves in vivo in future studies. Another limitation of this study is the absence of mechanistic studies using molecular tools or direct protein-level validation (e.g., Western blot or immunohistochemical investigations) due to funding constraints.

## Figures and Tables

**Table 1:** Formation constants of [Pd(DMEN)(H_2_O)_2_]^2+^ complex at 25 °C and 0.1 M ionic strength.

System	M H ^a^	logβ ^b^	pK_a_^c^
Pd(DMEN)-OH			
	1-1	−5.29 (0.02)	5.29
	1-2	−14.74 (0.02)	9.45
	2-1	−2.12 (0.07)	Kdimer = 3.17

^a^ M and H are the stoichiometric coefficients for Pd(DMEN), with the coefficient −1 indicating a proton loss; ^b^ log β pertains to Pd(DMEN)-OH. Standard deviations are indicated in brackets; the sum of squared residuals is below 5 × 10^7^; ^c^ denotes the pKa of the species or the aqua complex.

**Table 2:** Important optimized bond lengths (Å) and bond angles (°) of the complex [Pd(DMEN)*Cl*_2_].

Bond Lengths (Å)		Bond Angles (°)	
Pd-N1	2.108	N1-Pd-N2	84.37
Pd-N2	2.156	N1-Pd-Cl1	87.20
Pd-Cl1	2.386	N2-Pd-Cl2	92.86
Pd-Cl2	2.386	Cl1-Pd-Cl2	95.53
		N1-Pd-Cl2	176.6
		N2-Pd-Cl1	171.5
		N1-N2-Cl2-Cl1	−0.479 *

Note: * dihedral angle.

**Table 3:** Calculated energies and properties of DMEN and [Pd(DMEN)*Cl*_2_].

Property	DMEN	[Pd(DMEN)Cl_2_]
E (a.u.)	−269.216	−1316.430
HOMO (eV)	−5.9474	−6.3822
LUMO (eV)	−0.3260	−2.6656
E_g_ (eV)	5.6214	3.7166
Dipole moment (Debye)	1.3486	13.6537
I = −E_HOMO_	5.9474	6.3822
A = −E_LUMO_	0.326	2.6656
χ = (I + A)/2	3.1367	4.5239
η = (I − A)/2	2.8107	1.8583
S = 1/2η	0.1779	0.2691
μ = −χ	−3.1367	−4.5239
ω = μ2/2η	1.7503	5.5066

**Table 4:** The docking interaction data calculations of DMEN and [Pd(DMEN)Cl_2_] with the active sites of the receptor of liver cancer protein (PDB ID: 5A19).

	Receptor	Interaction	Distance (Å) *	E (kcal/mol)
DMEN				
N 1	NZ LYS 97	H-acceptor	3.07 (2.28)	−4.5
N 4	NH1 ARG 168	H-acceptor	3.38 (2.41)	−2.6
[Pd(DMEN)Cl_2_]				
N 1	OE1 GLU 57	H-donor	2.97 (2.23)	−4.0
PD 18	O THR 262	Metal	2.68	−0.6
N 1	OE1 GLU 57	Ionic	2.97	−4.7
N 1	OE2 GLU 57	Ionic	3.86	−0.8

Note: * lengths of H-bonds are in brackets.

**Table 5:** TGA mass loss of [Pd(DMEN)Cl_2_] complex in the temperature range ~25 to 700 °C with heating rate of 10 degree/min.

Assignment Loss	TGA °C	%Wt Loss Found (Calcd)
C_4_H_12_N_2_ + Cl	220–320	47.07 (46.56)
Cl	320–570	12.92 (13.35)
Remaining Pd	>570	40.01 (40.09)

**Table 6:** Effects of treatment of liver-cancer induced (DEN/TAA) rats with MNPs compounds on serum ALT and AST activities.

	AST (IU/L)	ALT (IU/L)
Negative control	31.94 ± 1.98 ^a^	134.73 ± 4.52 ^a^
DEN/TAA positive control	48.95 ± 0.66 ^c^	168.26 ± 0.54 ^b^
DEN/TAA +MNPs	32.72 ± 1.26 ^ab^	134.89 ± 7.80 ^a^
DEN/TAA + complex	32.81 ± 2.97 ^ab^	138.49 ± 8 ^a^
DEN/TAA + complex loaded MNPs	31.94 ± 0.00 ^a^	132.85 ± 11.91 ^a^

Results are presented as mean ± SEM. Means with different superscripts (a, b, c) between treatments in the same group are significantly different at *p* < 0.05.

**Table 7:** Effects of treatment of liver-cancer induced (DEN/TAA) rats with MNP compounds on serum TNF-α and MMP-9 levels.

Groups	TNF-α	MMP-9
Negative control	82.92 ± 0.64 ^a^	491.34 ± 48.74 ^a^
DEN/TAA control	125.97 ± 16.22 ^b^	1079.79 ± 124.93 ^bc^
DEN/TAA +MNPs	93.15 ± 8.20 ^ab^	638.53 ± 69.72 ^ab^
DEN/TAA + complex	102.8150 ± 6.94 ^ab^	2252.52 ± 407.29 ^d^
DEN/TAA + complex loaded MNPs	105.69 ± 10.43 ^ab^	1079.72 ± 0.00 ^bc^

Results are presented as mean ± SEM. Means with different superscripts (a, b, c, d) between treatments in the same group are significantly different at *p* < 0.05.

## Data Availability

Data will be made available upon request.

## References

[1] Sun T., Zhang Y.S., Pang B., Hyun D.C., Yang M., Xia Y. (2014). Engineered nanoparticles for drug delivery in cancer therapy. Chem. Int. Ed..

[2] Grigore M.E. (2017). Organic and inorganic nano-systems used in cancer treatment. J. Med. Res. Health Educ..

[3] Rosenberg B., VanCamp L., Trosko J.E., Mansour V.H. (1969). Platinum compounds: A new class of potent antitumour agents. Nature.

[4] Sung H., Ferlay J., Siegel R.L., Laversanne M., Soerjomataram I., Jemal A., Bray F. (2021). Global Cancer Statistics 2020: GLOBOCAN Estimates of Incidence and Mortality Worldwide for 36 Cancers in 185 Countries. CA A Cancer J. Clin..

[5] Akinyemiju T., Abera S., Ahmed M., Alam N., Alemayohu M.A., Allen C., Al-Raddadi R., Alvis-Guzman N., Amoako Y., Artaman A. (2017). The burden of primary liver cancer and underlying etiologies from 1990 to 2015 at the global, regional, and national level: Results from the global burden of disease study 2015. JAMA Oncol..

[6] Asrani S.K., Devarbhavi H., Eaton J., Kamath J., Hepatol P.S. (2019). Burden of liver diseases in the world. J. Hepatol..

[7] Dasari S., Tchounwou P.B. (2014). Cisplatin in cancer therapy: Molecular mechanisms of action. Eur. J. Pharmacol..

[8] Galluzzi L., Senovilla L., Vitale I., Michels J., Martins I., Kepp O., Castedo M., Kroemer G. (2012). Molecular mechanisms of cisplatin resistance. Oncogene.

[9] Zhao G., Rodriguez B.L. (2013). Molecular targeting of liposomal nanoparticles to tumor microenvironment. Int. J. Nanomed..

[10] Gutteridge W.E. (1985). Existing chemotherapy and its limitations. Br. Med. Bull..

[11] Wicki A., Witzigmann D., Balasubramanian V., Huwyler J. (2015). Nanomedicine in cancer therapy: Challenges, opportunities, and clinical applications. J. Control. Release.

[12] Mabrouk M., Abd El-Wahab R.M., Abo-Elfadl M.T., Beherei H.H., Selim M.M., Ibrahim A.M., Das D.B. (2022). Magnetic nanosystems substituted with zinc for enhanced antibacterial, drug delivery and cell viability behaviours. Colloids Surf. A Physicochem. Eng. Asp..

[13] Zoppellaro G. (2020). Iron Oxide Magnetic Nanoparticles (NPs)-Tailored for Biomedical Applications. Magnetic Nanoheterostructures: Diagnostic, Imaging and Treatment.

[14] Ansari L., Jaafari M.R., Bastami T.R., Malaekeh-Nikouei B. (2018). Improved anticancer efficacy of epirubicin by magnetic mesoporous silica nanoparticles: Invitro and in vivo studies. Artif. Cells Nanomed. Biotechnol..

[15] Orel V., Mitrelias T., Shevchenko A., Romanov A., Barnes C., Tselepi M., Burlaka A., Lukin S., Schepoyin I. (2013). Studies of Complex Magnetic Nanoparticles with Anticancer Agents for Cancer Therapy. J. Basic Appl. Phys..

[16] Mabrouk M., Ibrahim Fouad G., El-Sayed S.A.M., Rizk M.Z., Beherei H.H. (2022). Hepatotoxic and Neurotoxic Potential of Iron Oxide Nanoparticles in Wistar Rats: A Biochemical and Ultrastructural Study. Biol. Trace Elem. Res..

[17] Shehata M.R., Shoukry M.M., Mabrouk M.A., Van Eldik R. (2016). Synthesis, X-ray structure, DFT and thermodynamic studies of mono-and binuclear palladium (II) complexes involving 1,4-bis(2-hydroxyethyl) piperazine, bio-relevant ligands and 4, 4′-bipiperidine. J. Coord. Chem..

[18] Frisch M.J., Trucks G.W., Schlegel H.B., Scuseria G.E., Robb M.A., Cheeseman J.R., Scalmani G., Barone V., Petersson G.A., Nakatsuji H. (2016). Fox. Gaussian 09, Revision A.02.

[19] C.C.G. Inc (2022). “Molecular Operating Environment (MOE 2022.02)” Chemical Computing Group ULC, 1010 Sherbrooke St. West, Suite #910.

[20] Reitman S., Frankel S. (1957). In vitro determination of transaminase activity in serum. Am. J. Clin. Pathol..

[21] Zhu M., Diao G. (2011). Synthesis of porous Fe_3_O_4_ nanospheres and its application for the catalytic degradation of xylenol orange. Am. J. Phys. Chem. C.

[22] Shaker S., Zafarian S., Chakra C.S., Rao K.V. (2013). Preparation and characterization of magnetite nanoparticles by Sol-Gel method for water treatment. Int. J. Innov. Res. Sci. Eng. Technol..

[23] Molina M.M., Seabra A.B., de Oliveira M.G., Itri R., Haddad P.S. (2013). Nitric oxide donor superparamagnetic iron oxide nano-particles. Mater. Sci. Eng. C.

[24] Gonçalves L.C., Seabra A.B., Pelegrino M.T., De Araujo D.R., Bernardes J.S., Haddad P.S. (2017). Superparamagnetic iron oxide nanoparticles dispersed in Pluronic F127 hydrogel: Potential uses in topical applications. RSC Adv..

[25] Labani M.M., Rezaee R., Saeedi A., Al Hinai A. (2013). Evaluation of pore size spectrum of gas shale reservoirs using low pressure nitrogen adsorption, gas expansion and mercury porosimetry: A case study from the Perth and Canning Basins, Western Australia. J. Pet. Sci. Eng..

[26] Bodade A.B., Taiwade M.A., Chaudhari G.N. (2017). Bioelectrode based chitosan-nanocopperoxide for application to lipase biosensor. J. Appl. Pharm. Res..

[27] Kumar K.N., Padma R., Ratnakaram Y.C., Kang M. (2017). Bright green emission from f-MWCNT embedded co-doped Bi 3++ Tb 3+: Polyvinyl alcohol polymer nanocomposites for photonic applications. RSC Adv..

[28] Sridharan K., Endo T., Cho S.G., Kim J., Park T.J., Philip R. (2013). Single step synthesis and optical limiting properties of Ni–Ag and Fe–Ag bimetallic nanoparticles. Opt. Mater..

[29] Lin S., Fang J., Huang Y., Zhang Y., Zhong J., Xiang W., Liang X. (2017). Study on the iron oxide/glass nanocomposite materials: Fabrication, microstructure and ultrafast nonlinear optical properties. J. Mater. Sci. Mater. Electron..

[30] Fujii T., Sawatzky G.A., Voogt F.C., Hibma T., Okada K. (1999). In situ XPS analysis of various iron oxide films grown by NO_2_-assisted molecular-beam epitaxy. Phys. Rev. B.

[31] Huang C., Zhang H., Sun Z., Zhao Y., Chen S., Tao R., Liu Z. (2011). Porous Fe_3_O_4_ nanoparticles: Synthesis and application in catalyzing epoxidation of styrene. J. Colloid Interface Sci..

[32] Rao P.M., Zheng X. (2011). Unique Magnetic Properties of Single Crystal γ-Fe_2_O_3_ Nanowires Synthesized by Flame Vapor Deposition. Nano Lett..

[33] Cao D., Li H., Pan L., Li J., Wang X., Jing P., Liu Q. (2016). High saturation magnetization of γ-Fe_2_O_3_ nano-particles by a facile one-step synthesis approach. Sci. Rep..

[34] Yu Y., Fan S.S., Wang X., Ma Z.W., Dai H.W., Han J.B. (2014). Large third-order optical nonlinearity in coupled Au–Ni–Au composite nanorods. Mater. Lett..

[35] C.C.G. ULC (2020). Molecular Operating Environment (MOE), 2020.09, 1010 Sherbrooke St. West, Suite #910.

[36] Asl A.M., Abdouss M., Kalaee M.R., Homami S.S., Pourmadadi M. (2024). Targeted delivery of quercetin using gelatin/starch/Fe_3_O_4_ nanocarrier to suppress the growth of liver cancer HepG_2_ cells. Int. J. Biol. Macromol..

[37] Castañeda C., Alvarado I., Martínez J.J., Brijaldo M.H., Passosc F.B., Rojas H. (2019). Enhanced photocatalytic reduction of 4-nitrophenol over Ir/CeO_2_ photocatalysts under UV irradiation. J. Chem. Technol. Biotechnol..

[38] Xu Y.Y., Zhao D., Zhang X.J., Jin W.T., Kashkarov P., Zhang H. (2009). Synthesis and characterization of single-crystalline α-Fe_2_O_3_ nanoleaves. Phys. E Low Dimens. Syst. Nanostruct..

[39] Rice E.W., Baird R.B., Eaton A.D., Clesceri L.S., American Public Health Association (APHA), American Water Works Association (AWWA), Water Environment Federation (WEF) (2017). Standard Methods for the Examination of Water and Wastewater.

[40] Gaharwar U.S., Meena R., Rajamani P. (2017). Iron oxide nanoparticles induced cytotoxicity, oxidative stress and DNA damage in lymphocytes. J. Appl. Toxicol..

[41] Krishnamurthy C., Keshavayya J., Maliyappa M.R., Shekharagouda P. (2024). Synthesis of anthraquinone-based azo-metal(II) complexes as potent anti-oxidizing agents. J. Coord. Chem..

[42] Pasdar H., Hedayati S.B., Foroughifar N., Davallo M. (2017). Synthesis, Characterization and Antibacterial Activity of Novel 1,3-Diethyl-1,3-bis(4-nitrophenyl)urea and Its Metal(II) Complexes. Molecules.

[43] Shoukry M.M., van Eldik R. (2023). Equilibrium Studies on Pd(II)-Amine Complexes with Bio-Relevant Ligands in Reference to Their Antitumor Activity. Int. J. Mol. Sci..

[44] Godymchuk A., Papina I., Karepina E., Kuznetsov D., Lapin I., Svetlichnyi V. (2019). Agglomeration of iron oxide nanoparticles: pH effect is stronger than amino acid acidity. J. Nanopart. Res..

[45] Hidekazu T. (2022). Influence of Anions and Cations on the Formation of Iron Oxide Nanoparticles in Aqueous Media. Kona Powder Part. J..

[46] Shehata M.R., Shoukry M.M., Ali S. (2012). Thermodynamics of the interaction of Pd (DMEN) (H_2_O) 22+ with bio-relevant ligands with reference to the deactivation of metal-based drug by thiol ligands. Spectrochim. Acta Part A Mol. Biomol. Spectrosc..

[47] Zhu X., Gu J., Li Y., Zhao W., Shi J. (2014). Magnetic core-mesoporous shell nanocarriers with drug anchorages suspended in mesopore interior for cisplatin delivery. Microporous Mesoporous Mater..

[48] Sallem F., Haji R., Vervandier-Fasseur D., Nury T., Maurizi L., Boudon J., Millot N. (2019). Elaboration of trans-resveratrol derivative-loaded superparamagnetic iron oxide nanoparticles for glioma treatment. Nanomaterials.

[49] Manjili H.K., Ma’mani L., Izadi A., Moslemi E., Mashhadikhan M., Mohammadi M.M., Naderi-Manesh H. (2015). Sulforaphane loaded PEGylated iron oxide-gold core shell nanoparticles: A promising delivery system for cancer therapy. Am. Int. J. Contemp. Sci. Res..

[50] Kheiri Manjili H., Ma’mani L., Tavaddod S., Mashhadikhan M., Shafiee A., Naderi-Manesh H.D. (2016). L-sulforaphane loaded Fe_3_O_4_@ gold core shell nanoparticles: A potential sulforaphane delivery system. PLoS ONE.

[51] Saadat M., Mostafaei F., Mahdinloo S., Abdi M., Zahednezhad F., Zakeri-Milani P., Valizadeh H. (2021). Drug delivery of pH-Sensitive nanoparticles into the liver cancer cells. J. Drug Deliv. Sci. Technol..

[52] Ebadi M., Bullo S., Buskara K., Hussein M.Z., Fakurazi S., Pastorin G. (2020). Release of a liver anticancer drug, sorafenib from its PVA/LDH-and PEG/LDH-coated iron oxide nanoparticles for drug delivery applications. Sci. Rep..

[53] Ding Y.F., Wu Z.H., Wei Y.J., Shu L., Peng Y.R. (2017). Hepatic inflammation-fibrosis-cancer axis in the rat hepatocellular carcinoma induced by diethylnitrosamine. J. Cancer Res. Clin. Oncol..

[54] Abood W.N., Bradosty S.W., Shaikh F.K., Salehen N.A., Farghadani R., Agha N.F.S., Al-Medhtiy M.H., Kamil T.D.A., Agha A.S., Abdulla M.A. (2020). Garcinia mangostana peel extracts exhibit hepatoprotective activity against thioacetamide-induced liver cirrhosis in rats. J. Funct. Foods.

[55] Li G., Qi L., Chen H., Tian G. (2022). Involvement of NF-κB/PI3K/AKT signaling pathway in the protective effect of prunetin against a diethylnitrosamine induced hepatocellular carcinogenesis in rats. J. Biochem. Mol. Toxicol..

[56] Liu J., Wen X., Liu B., Zhang Q., Zhang J., Miao H., Zhu R. (2016). Diosmetin inhibits the metastasis of hepatocellular carcinoma cells by downregulating the expression levels of MMP-2 and MMP-9. Mol. Med. Rep..

[57] Rashid Z.A., Bardaweel S.K. (2023). Novel matrix metalloproteinase-9 (MMP-9) inhibitors in cancer treatment. Int. J. Mol. Sci..

[58] Hidalgo M., Eckhardt S.G. (2001). Development of matrix metalloproteinase inhibitors in cancer therapy. J. Natl. Cancer Inst..

[59] Abdel-Hamid N.M., Abass S.A., Eldomany R.A., Abdel-Kareem M.A., Zakaria S. (2022). Dual regulating of mitochondrial fusion and Timp-3 by leflunomide and diallyl disulfide combination suppresses diethylnitrosamine-induced hepatocellular tumorigenesis in rats. Life Sci..

[60] Kurma K., Manches O., Chuffart F., Sturm N., Gharzeddine K., Zhang J., Decaens T. (2021). DEN-induced rat model reproduces key features of human hepatocellular carcinoma. Cancers.

[61] Singh D., Singh M., Yadav E., Falls N., Dangi D.S., Kumar V., Verma A. (2018). Attenuation of diethylnitrosamine (DEN)–Induced hepatic cancer in experimental model of Wistar rats by *Carissa carandas* embedded silver nanoparticles. Biomed. Pharmacother..

[62] Santos N.P., Pereira I.C., Pires M.J., Lopes C., Andrade R., Oliveira M.M., Oliveira P.A. (2022). Histology, bioenergetics and oxidative stress in mouse liver exposed to N-diethylnitrosamine. In Vivo.

[63] Ojeaburu S.I., Oriakhi K. (2021). Hepatoprotective, antioxidant and, anti-inflammatory potentials of gallic acid in carbon tetrachloride-induced hepatic damage in Wistar rats. Toxicol. Rep..

